# 17-Deoxoestrone [estra-1,3,5(10)-trien-3-ol]–methanol (3/1)

**DOI:** 10.1107/S1600536811013651

**Published:** 2011-04-16

**Authors:** Kamal Aziz Ketuly, A. Hamid A. Hadi, Seik Weng Ng, Edward R. T. Tiekink

**Affiliations:** a Department of Chemistry, University of Malaya, 50603 Kuala Lumpur, Malaysia

## Abstract

Three independent mol­ecules of the title estrone derivative and a mol­ecule of methanol comprise the asymmetric unit of the title compound [systematic name: 13-methyl-6,7,8,9,11,12,13,14,15,16-deca­hydro­cyclo­penta­[*a*]phenanthren-3-ol–meth­an­ol (3/1)], 3C_18_H_24_O·CH_3_OH. Two of the estrone mol­ecules exhibit 50:50 disorder (one displays whole-mol­ecule disorder and the other partial disorder in the fused five- and six-membered rings) so that five (partial) mol­ecular conformations are discernable. The conformation of the six-membered ring abutting the aromatic ring is close to a half-chair in all five components. The conformation of the six-membered ring fused to the five-membered ring is based on a chair with varying degrees of distortion ranging from minor to significant. Two distinct conformations are found for the five-membered ring: in four mol­ecules, the five-membered ring is twisted about the bond linking it to the six-membered ring, and in the other, the five-membered ring is an envelope with the quaternary C atom being the flap atom. The crystal packing features O—H⋯O hydrogen bonding whereby the four mol­ecules comprising the asymmetric unit are linked into a supra­molecular chain along the *b* axis.

## Related literature

For the original synthesis, see: Huang-Minlon (1949 ▶). For geometric and structural features for a series of cholestane derivatives, see: Rajnikant *et al.* (2006 ▶). For background to steroidal estrogen boronic acids and boronates, see: Ketuly & Hadi (2010 ▶). For related structures see: Ketuly *et al.* (1997 ▶, 2010 ▶).
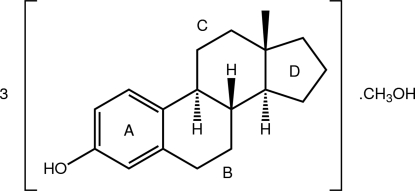


## Experimental

### 

#### Crystal data

3C_18_H_24_O·CH_4_O
*M* *_r_* = 801.16
Monoclinic, 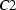

*a* = 24.3084 (16) Å
*b* = 7.7235 (5) Å
*c* = 26.6479 (18) Å
β = 114.292 (1)°
*V* = 4560.1 (5) Å^3^
*Z* = 4
Mo *K*α radiation
μ = 0.07 mm^−1^
*T* = 100 K
0.35 × 0.20 × 0.10 mm

#### Data collection

Bruker SMART APEX CCD diffractometer
Absorption correction: multi-scan (*SADABS*; Sheldrick, 1996 ▶) *T*_min_ = 0.976, *T*_max_ = 0.993
21707 measured reflections
5612 independent reflections
4481 reflections with *I* > 2σ(*I*)
*R*_int_ = 0.050

#### Refinement

*R*[*F* ^2^ > 2σ(*F* ^2^)] = 0.052
*wR*(*F* ^2^) = 0.141
*S* = 1.02
5612 reflections
616 parameters
234 restraints
H-atom parameters constrained
Δρ_max_ = 0.37 e Å^−3^
Δρ_min_ = −0.33 e Å^−3^

### 

Data collection: *APEX2* (Bruker, 2009 ▶); cell refinement: *SAINT* (Bruker, 2009 ▶); data reduction: *SAINT*; program(s) used to solve structure: *SHELXS97* (Sheldrick, 2008 ▶); program(s) used to refine structure: *SHELXL97* (Sheldrick, 2008 ▶); molecular graphics: *ORTEP-3* (Farrugia, 1997 ▶), *DIAMOND* (Brandenburg, 2006 ▶) and *QMOL* (Gans & Shalloway, 2001 ▶); software used to prepare material for publication: *publCIF* (Westrip, 2010 ▶).

## Supplementary Material

Crystal structure: contains datablocks global, I. DOI: 10.1107/S1600536811013651/hb5844sup1.cif

Structure factors: contains datablocks I. DOI: 10.1107/S1600536811013651/hb5844Isup2.hkl

Additional supplementary materials:  crystallographic information; 3D view; checkCIF report

## Figures and Tables

**Table 1:** Hydrogen-bond geometry (Å, °)

*D*—H⋯*A*	*D*—H	H⋯*A*	*D*⋯*A*	*D*—H⋯*A*
O1—H1⋯O3	0.84	1.83	2.662 (3)	170
O2—H2⋯O4	0.84	1.82	2.655 (4)	170
O3—H3⋯O2	0.84	1.88	2.711 (3)	173
O4—H4⋯O1^i^	0.84	1.90	2.736 (4)	178

Symmetry code: (i) 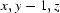.

**Fig. 1.**
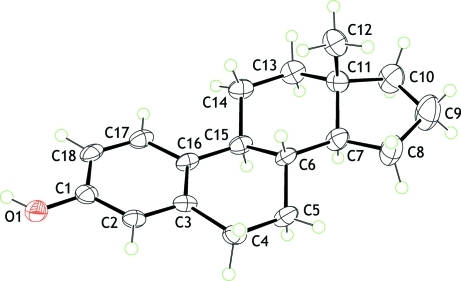
The molecular structures of the first independent molecule comprising the asymmetric unit of (I) showing displacement ellipsoids at the 50% probability level.

**Fig. 2.**
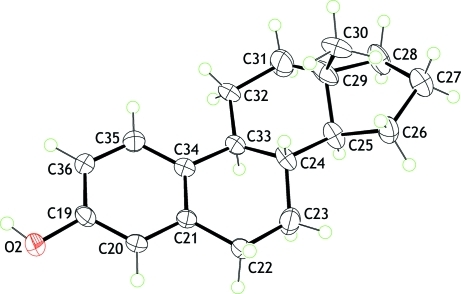
The molecular structures of the second independent molecule comprising the asymmetric unit of (I) showing displacement ellipsoids at the 50% probability level.

**Fig. 3.**
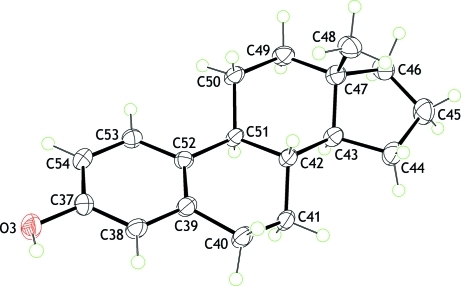
The molecular structures of the third independent molecule comprising the asymmetric unit of (I) showing displacement ellipsoids at the 50% probability level.

**Fig. 4.**
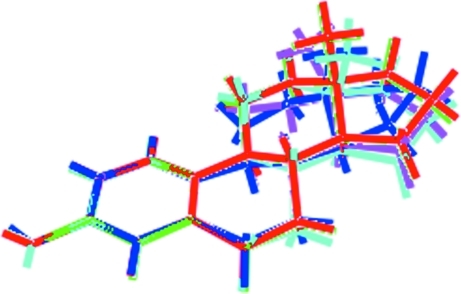
Overlay diagram the three independent molecules comprising the asymmetric unit of (I) and their disordered components. The disordered components of the molecule shown in Fig. 1 are shown in red and green, the disordered components of Fig. 2 are shown in blue and pink, and the molecule in Fig. 3 is shown in light-blue.

**Fig. 5.**
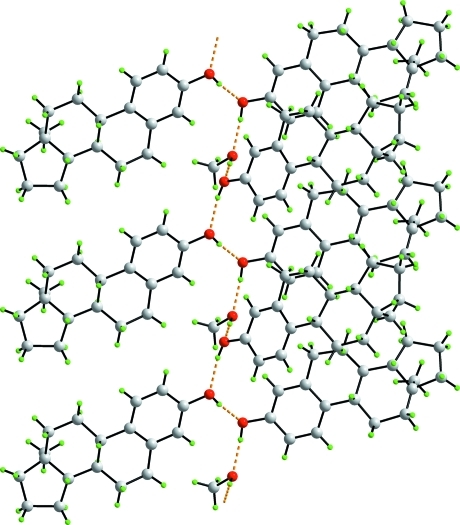
View of the supramolecular chain aligned along the b axis of the crystal structure of (I). The O—H···O contacts hydrogen bonds are shown as orange dashed lines. Only one orientation of the disordered O—H1 atom is shown.

## supplementary crystallographic information

### Comment

The title estrone, (I), has been prepared by a modified procedure from Huang-Minlon (1949) as a precursor for the synthesis of steroidal estrogen boronic acids and boronates (Ketuly & Hadi, 2010). Related structures have been reported (Ketuly *et al.*, 1997; Ketuly *et al.*, 2010) as has a systematic evaluation of geometric and structural features for a series of cholestane derivatives (Rajnikant *et al.*, 2006). Herein, the crystal and molecular structure of (I) is described.

Three independent estrone molecules, Figs 1 - 3, and a solvent methanol molecule comprise the asymmetric unit of (I). The first independent molecule exhibits whole molecule disorder pivoted on the O1 atom. As seen from the overlay diagram, Fig. 4, the two components of equal weight, *i.e*. shown as red and green, have almost identical conformations. Referring to the Scheme for numbering of the rings, the six-membered ring B, being fused to an aromatic ring, has an half-chair conformation, six-membered ring C has a slightly twisted chair conformation, and five-membered ring C is twisted on the bond linking it to the six-membered ring. Disorder is also evident in the second independent molecule. In this case, the 50:50 disorder is restricted to rings C and D. As shown in pink and blue in Fig. 4, the rings have distinct conformations. For the molecule illustrated in pink, ring B is an half-chair and the conformation of ring C is that of a twisted chair. Ring D is an envelope on the quaternary-C29 atom. In the second disordered conformation, ring B is an half-chair and ring C is a slightly twisted chair. Ring D is distinct from that in the first orientation in that is adopts a twisted conformation about the C25—C29 bond. The third independent molecule, shown as light-blue in Fig. 4, is different from the other molecules in that ring B is a slightly twisted half-chair, ring C is a slightly twisted chair, and ring C is twisted about the C43—C47 bond.

In the crystal packing, the four independent molecules are connected *via* a sequence of O—H···O hydrogen bonds, Table 1. These link molecules into a supramolecular chain along the *b* axis, Fig. 5.

### Experimental

The title compound (I) was prepared by a modified procedure from Huang-Minlon (1949) Estrone (1.5 g) was dissolved in diethylene glycol (40 ml), with heating and stirring in an oil bath at 388–393 K. Hydrazine hydrate (98–100%) was added in four 3 ml portions at 15 min intervals. During this time stirring and heating was continued and the flask connected to a drying tube. While the temperature was raised to 423 K, nitrogen was bubbled through the reaction mixture to remove excess reagent and water as by-product. When most of the reagent had been evaporated, KOH pellets (9.6 g) were added. The flask was then fitted with a 50 cm air condenser (with a drying tube), and the reaction mixture was refluxed for 2 h under nitrogen. The cooled basic reaction mixture was diluted with water and extracted three times with ether, and each extract was washed five times with water. The ether extracts were dried under vacuum and the residue crystallized from MeOH/water, yielding colourless crystals (1.305 g, 91%), *M*.pt. 401–403 K. This product was recrystallized three times from MeOH/water, yielding (I). *M*.pt. 407–408 K, (Lit. 407–407.5 K (Huang-Minlon, 1949)).

### Refinement

Carbon-bound H-atoms were placed in calculated positions (C–H 0.95 to 0.98 Å) and were included in the refinement in the riding model approximation, with *U*_iso_(H) set to 1.2 to 1.5*U*_eq_(C). The hydroxy H-atoms were similarly placed (O—H 0.84 Å) and their *U*_iso_(H) similarly tied. Of the three independent molecules, one (with O1) is whole-molecule disordered and another (with O2) is partially disordered. The disorder was assumed to be a 1:1 disorder as the occupancy could not be refined. The aromatic C–C distances were restrained to 1.39±0.01 Å and the aliphatic ones to 1.54±0.01 Å; for the aliphatic arbon atoms, the 1,3-related distances were restrained to 2.52±0.01 Å. The anisotropic displacement factors of the primed atoms were restrained to be equal to those of the unprimed ones, and the anisotropic displacement factors of the disordered carbon atoms were restrained to be nearly isotropic. In the absence of significant anomalous scattering effects, 2199 Friedel pairs were averaged in the final refinement. However, the absolute configuration was assigned on the basis of the known chirality of the estrone starting material.

### Crystal data

**Table:**

3C_18_H_24_O·CH_4_O	*F*(000) = 1752
*M**_r_* = 801.16	*D*_x_ = 1.167 Mg m^−^^3^
Monoclinic, *C*2	Mo *K*α radiation, λ = 0.71073 Å
Hall symbol: C 2y	Cell parameters from 5587 reflections
*a* = 24.3084 (16) Å	θ = 2.3–28.2°
*b* = 7.7235 (5) Å	µ = 0.07 mm^−^^1^
*c* = 26.6479 (18) Å	*T* = 100 K
β = 114.292 (1)°	Block, colourless
*V* = 4560.1 (5) Å^3^	0.35 × 0.20 × 0.10 mm
*Z* = 4	

### Data collection

**Table:**

Bruker SMART APEX CCD diffractometer	5612 independent reflections
Radiation source: fine-focus tube	4481 reflections with *I* > 2σ(*I*)
Mirror	*R*_int_ = 0.050
ω scans	θ_max_ = 27.5°, θ_min_ = 1.9°
Absorption correction: multi-scan (*SADABS*; Sheldrick, 1996)	*h* = −31→31
*T*_min_ = 0.976, *T*_max_ = 0.993	*k* = −10→10
21707 measured reflections	*l* = −34→34

### Refinement

**Table:**

Refinement on *F*^2^	Secondary atom site location: difference Fourier map
Least-squares matrix: full	Hydrogen site location: inferred from neighbouring sites
*R*[*F*^2^ > 2σ(*F*^2^)] = 0.052	H-atom parameters constrained
*wR*(*F*^2^) = 0.141	*w* = 1/[σ^2^(*F*_o_^2^) + (0.0693*P*)^2^ + 3.2841*P*] where *P* = (*F*_o_^2^ + 2*F*_c_^2^)/3
*S* = 1.02	(Δ/σ)_max_ = 0.001
5612 reflections	Δρ_max_ = 0.37 e Å^−^^3^
616 parameters	Δρ_min_ = −0.33 e Å^−^^3^
234 restraints	Absolute structure: 2199 Friedel pairs were merged
Primary atom site location: structure-invariant direct methods	

### Special details

**Table:**

Geometry. All e.s.d.'s (except the e.s.d. in the dihedral angle between two l.s. planes) are estimated using the full covariance matrix. The cell e.s.d.'s are taken into account individually in the estimation of e.s.d.'s in distances, angles and torsion angles; correlations between e.s.d.'s in cell parameters are only used when they are defined by crystal symmetry. An approximate (isotropic) treatment of cell e.s.d.'s is used for estimating e.s.d.'s involving l.s. planes.

### Fractional atomic coordinates and isotropic or equivalent isotropic displacement parameters (Å^2^)

**Table:**

	*x*	*y*	*z*	*U*_iso_*/*U*_eq_	Occ. (<1)
O1	0.53469 (10)	1.0006 (3)	0.20950 (10)	0.0371 (6)	
H1	0.5204	0.9020	0.1982	0.056*	0.50
H1'	0.5212	0.8991	0.2057	0.056*	0.50
O2	0.57490 (9)	0.4963 (3)	0.14534 (10)	0.0321 (5)	
H2	0.5701	0.3911	0.1366	0.048*	
O3	0.50222 (10)	0.6730 (3)	0.18194 (10)	0.0351 (6)	
H3	0.5235	0.6106	0.1711	0.053*	
O4	0.55238 (11)	0.1609 (4)	0.12576 (11)	0.0421 (6)	
H4	0.5466	0.1134	0.1515	0.063*	
C1	0.5807 (19)	1.0199 (18)	0.2620 (12)	0.0308 (17)	0.50
C2	0.6019 (7)	1.1867 (17)	0.2777 (4)	0.0258 (19)	0.50
H2A	0.5825	1.2797	0.2535	0.031*	0.50
C3	0.6503 (4)	1.2235 (10)	0.3273 (3)	0.0243 (15)	0.50
C4	0.6678 (3)	1.4130 (9)	0.3394 (3)	0.0302 (12)	0.50
H4A	0.6321	1.4799	0.3372	0.036*	0.50
H4B	0.6803	1.4586	0.3110	0.036*	0.50
C5	0.7192 (3)	1.4396 (9)	0.3963 (3)	0.0347 (11)	0.50
H5A	0.7392	1.5518	0.3970	0.042*	0.50
H5B	0.7023	1.4441	0.4244	0.042*	0.50
C6	0.7658 (3)	1.2950 (8)	0.4109 (3)	0.0271 (10)	0.50
H6	0.7798	1.2834	0.3806	0.032*	0.50
C7	0.8204 (3)	1.3310 (9)	0.4649 (3)	0.0346 (11)	0.50
H7	0.8048	1.3326	0.4942	0.041*	0.50
C8	0.8559 (4)	1.4986 (10)	0.4715 (3)	0.0503 (15)	0.50
H8A	0.8349	1.5973	0.4796	0.060*	0.50
H8B	0.8622	1.5247	0.4378	0.060*	0.50
C9	0.9164 (5)	1.4603 (12)	0.5206 (4)	0.066 (2)	0.50
H9A	0.9204	1.5314	0.5528	0.079*	0.50
H9B	0.9507	1.4866	0.5109	0.079*	0.50
C10	0.9153 (4)	1.2642 (10)	0.5336 (3)	0.0495 (15)	0.50
H10A	0.9553	1.2108	0.5428	0.059*	0.50
H10B	0.9040	1.2469	0.5649	0.059*	0.50
C11	0.8676 (3)	1.1855 (9)	0.4805 (2)	0.0324 (11)	0.50
C12	0.8956 (4)	1.1604 (13)	0.4388 (3)	0.0412 (16)	0.50
H12A	0.9236	1.0623	0.4502	0.062*	0.50
H12B	0.9175	1.2656	0.4374	0.062*	0.50
H12C	0.8636	1.1375	0.4023	0.062*	0.50
C13	0.8376 (3)	1.0165 (10)	0.4873 (3)	0.0391 (12)	0.50
H13A	0.8266	1.0278	0.5191	0.047*	0.50
H13B	0.8666	0.9197	0.4950	0.047*	0.50
C14	0.7801 (3)	0.9748 (9)	0.4348 (3)	0.0332 (11)	0.50
H14A	0.7923	0.9434	0.4047	0.040*	0.50
H14B	0.7598	0.8731	0.4421	0.040*	0.50
C15	0.7352 (3)	1.1254 (8)	0.4155 (3)	0.0291 (10)	0.50
H15	0.7199	1.1437	0.4446	0.035*	0.50
C16	0.6799 (3)	1.0863 (8)	0.3618 (3)	0.0252 (12)	0.50
C17	0.6585 (4)	0.9186 (9)	0.3464 (4)	0.0316 (14)	0.50
H17	0.6773	0.8256	0.3708	0.038*	0.50
C18	0.6104 (6)	0.8834 (16)	0.2964 (5)	0.032 (2)	0.50
H18A	0.5980	0.7674	0.2859	0.038*	0.50
C1'	0.5807 (19)	1.051 (2)	0.2582 (12)	0.0308 (17)	0.50
C2'	0.5974 (7)	1.2241 (16)	0.2647 (5)	0.0258 (19)	0.50
H2'	0.5777	1.3046	0.2359	0.031*	0.50
C3'	0.6434 (4)	1.2783 (9)	0.3139 (3)	0.0243 (15)	0.50
C4'	0.6605 (3)	1.4689 (9)	0.3177 (3)	0.0302 (12)	0.50
H4'A	0.6236	1.5397	0.3080	0.036*	0.50
H4'B	0.6772	1.4936	0.2902	0.036*	0.50
C5'	0.7070 (3)	1.5246 (9)	0.3748 (3)	0.0347 (11)	0.50
H5'A	0.7273	1.6323	0.3714	0.042*	0.50
H5'B	0.6862	1.5484	0.3992	0.042*	0.50
C6'	0.7538 (3)	1.3821 (8)	0.3999 (2)	0.0271 (10)	0.50
H6'	0.7712	1.3506	0.3731	0.032*	0.50
C7'	0.8054 (3)	1.4399 (9)	0.4540 (2)	0.0346 (11)	0.50
H7'	0.7870	1.4628	0.4807	0.041*	0.50
C8'	0.8418 (4)	1.6017 (11)	0.4538 (3)	0.0503 (15)	0.50
H8'A	0.8191	1.7086	0.4534	0.060*	0.50
H8'B	0.8523	1.6022	0.4217	0.060*	0.50
C9'	0.8987 (5)	1.5852 (12)	0.5081 (3)	0.066 (2)	0.50
H9'1	0.9355	1.6033	0.5014	0.079*	0.50
H9'2	0.8981	1.6724	0.5351	0.079*	0.50
C10'	0.8980 (4)	1.4011 (11)	0.5298 (3)	0.0495 (15)	0.50
H10C	0.8841	1.4027	0.5599	0.059*	0.50
H10D	0.9387	1.3487	0.5437	0.059*	0.50
C11'	0.8534 (3)	1.2992 (9)	0.4799 (3)	0.0324 (11)	0.50
C12'	0.8849 (4)	1.2449 (12)	0.4428 (4)	0.0412 (16)	0.50
H12D	0.9143	1.1534	0.4610	0.062*	0.50
H12E	0.9057	1.3450	0.4360	0.062*	0.50
H12F	0.8548	1.2015	0.4077	0.062*	0.50
C13'	0.8224 (3)	1.1431 (9)	0.4919 (3)	0.0391 (12)	0.50
H13C	0.8076	1.1752	0.5201	0.047*	0.50
H13D	0.8521	1.0485	0.5071	0.047*	0.50
C14'	0.7688 (3)	1.0772 (9)	0.4399 (3)	0.0332 (11)	0.50
H14C	0.7844	1.0282	0.4140	0.040*	0.50
H14D	0.7479	0.9834	0.4504	0.040*	0.50
C15'	0.7235 (3)	1.2217 (8)	0.4109 (3)	0.0291 (10)	0.50
H15'	0.7047	1.2577	0.4364	0.035*	0.50
C16'	0.6717 (3)	1.1612 (9)	0.3566 (3)	0.0252 (12)	0.50
C17'	0.6509 (4)	0.9919 (9)	0.3498 (4)	0.0316 (14)	0.50
H17'	0.6689	0.9127	0.3793	0.038*	0.50
C18'	0.6048 (6)	0.9332 (16)	0.3013 (5)	0.032 (2)	0.50
H18'	0.5903	0.8175	0.2978	0.038*	0.50
C19	0.63506 (13)	0.5273 (5)	0.17821 (14)	0.0312 (7)	
C20	0.65239 (13)	0.6954 (4)	0.19521 (13)	0.0263 (7)	
H20	0.6233	0.7856	0.1825	0.032*	
C21	0.71149 (14)	0.7350 (4)	0.23044 (13)	0.0265 (7)	
C22	0.72835 (14)	0.9220 (5)	0.24470 (14)	0.0298 (7)	
H22A	0.7000	0.9733	0.2587	0.036*	
H22B	0.7231	0.9848	0.2106	0.036*	
C23	0.79207 (15)	0.9501 (5)	0.28701 (16)	0.0417 (9)	
H23A	0.7925	0.9421	0.3243	0.050*	
H23B	0.8051	1.0684	0.2826	0.050*	
C24	0.83713 (12)	0.8193 (5)	0.28247 (13)	0.0291 (7)	
H24	0.8366	0.8249	0.2448	0.035*	0.50
H24'	0.8341	0.8233	0.2439	0.035*	0.50
C25	0.9010 (3)	0.8549 (10)	0.3256 (5)	0.0329 (9)	0.50
H25	0.9031	0.8668	0.3637	0.039*	0.50
C26	0.9332 (3)	1.0042 (10)	0.3095 (3)	0.0375 (16)	0.50
H26A	0.9236	1.1166	0.3219	0.045*	0.50
H26B	0.9197	1.0082	0.2691	0.045*	0.50
C27	1.0029 (3)	0.9685 (11)	0.3383 (4)	0.0450 (17)	0.50
H27A	1.0235	1.0554	0.3673	0.054*	0.50
H27B	1.0212	0.9700	0.3113	0.054*	0.50
C28	1.0068 (6)	0.7803 (13)	0.3641 (5)	0.0421 (13)	0.50
H28A	1.0085	0.7846	0.4019	0.051*	0.50
H28B	1.0414	0.7130	0.3641	0.051*	0.50
C29	0.9437 (3)	0.7076 (11)	0.3205 (4)	0.0427 (12)	0.50
C30	0.9483 (3)	0.7570 (11)	0.2639 (3)	0.0380 (14)	0.50
H30A	0.9750	0.6748	0.2569	0.057*	0.50
H30B	0.9645	0.8744	0.2666	0.057*	0.50
H30C	0.9080	0.7519	0.2336	0.057*	0.50
C31	0.9289 (4)	0.5501 (11)	0.3333 (5)	0.0707 (16)	0.50
H31A	0.9440	0.5444	0.3739	0.085*	0.50
H31B	0.9517	0.4628	0.3223	0.085*	0.50
C32	0.8617 (3)	0.4922 (9)	0.3089 (4)	0.0390 (17)	0.50
H32A	0.8538	0.4186	0.2762	0.047*	0.50
H32B	0.8546	0.4210	0.3365	0.047*	0.50
C33	0.81816 (14)	0.6413 (5)	0.29238 (16)	0.0374 (8)	
H33	0.8114	0.6600	0.3265	0.045*	0.50
H33'	0.8201	0.6330	0.3305	0.045*	0.50
C34	0.75458 (15)	0.6010 (5)	0.25019 (16)	0.0379 (8)	
C35	0.73591 (17)	0.4339 (5)	0.2302 (2)	0.0539 (12)	
H35	0.7649	0.3432	0.2414	0.065*	
C36	0.67717 (16)	0.3952 (5)	0.19488 (18)	0.0462 (10)	
H36	0.6659	0.2800	0.1823	0.055*	
C37	0.44552 (13)	0.6013 (4)	0.16512 (13)	0.0269 (7)	
C38	0.43350 (13)	0.4347 (4)	0.14478 (13)	0.0267 (7)	
H38	0.4648	0.3682	0.1416	0.032*	
C39	0.37618 (14)	0.3624 (4)	0.12873 (13)	0.0252 (6)	
C40	0.36749 (14)	0.1753 (4)	0.10990 (14)	0.0299 (7)	
H40A	0.4026	0.1064	0.1345	0.036*	
H40B	0.3659	0.1685	0.0722	0.036*	
C41	0.30981 (13)	0.0976 (4)	0.11006 (13)	0.0256 (6)	
H41A	0.3019	−0.0169	0.0917	0.031*	
H41B	0.3142	0.0818	0.1484	0.031*	
C42	0.25784 (13)	0.2197 (4)	0.07961 (12)	0.0215 (6)	
H42	0.2593	0.2513	0.0438	0.026*	
C43	0.19548 (13)	0.1471 (4)	0.06774 (13)	0.0241 (6)	
H43	0.1931	0.1328	0.1041	0.029*	
C44	0.17621 (15)	−0.0260 (5)	0.03742 (15)	0.0337 (8)	
H44A	0.1941	−0.1246	0.0626	0.040*	
H44B	0.1883	−0.0332	0.0063	0.040*	
C45	0.10627 (15)	−0.0241 (5)	0.01659 (15)	0.0375 (8)	
H45A	0.0868	−0.0545	−0.0230	0.045*	
H45B	0.0932	−0.1085	0.0375	0.045*	
C46	0.08903 (14)	0.1616 (5)	0.02594 (14)	0.0323 (7)	
H46A	0.0530	0.2018	−0.0062	0.039*	
H46B	0.0804	0.1669	0.0591	0.039*	
C47	0.14434 (14)	0.2722 (4)	0.03357 (13)	0.0263 (7)	
C48	0.14362 (15)	0.3073 (5)	−0.02335 (13)	0.0330 (8)	
H48A	0.1806	0.3688	−0.0191	0.050*	
H48B	0.1084	0.3785	−0.0450	0.050*	
H48C	0.1414	0.1972	−0.0424	0.050*	
C49	0.15223 (13)	0.4399 (4)	0.06586 (14)	0.0295 (7)	
H49A	0.1446	0.4173	0.0990	0.035*	
H49B	0.1220	0.5252	0.0428	0.035*	
C50	0.21556 (13)	0.5173 (4)	0.08362 (14)	0.0267 (7)	
H50A	0.2198	0.6172	0.1082	0.032*	
H50B	0.2203	0.5606	0.0507	0.032*	
C51	0.26593 (13)	0.3857 (4)	0.11363 (12)	0.0212 (6)	
H51	0.2615	0.3518	0.1480	0.025*	
C52	0.32922 (12)	0.4613 (4)	0.13156 (12)	0.0214 (6)	
C53	0.34287 (13)	0.6289 (4)	0.15313 (12)	0.0236 (6)	
H53	0.3118	0.6967	0.1563	0.028*	
C54	0.40010 (14)	0.6995 (4)	0.17005 (12)	0.0261 (6)	
H54	0.4082	0.8133	0.1848	0.031*	
C55	0.51180 (17)	0.0929 (7)	0.07527 (17)	0.0532 (11)	
H55A	0.4705	0.1248	0.0692	0.080*	
H55B	0.5155	−0.0335	0.0759	0.080*	
H55C	0.5211	0.1399	0.0455	0.080*	
C25'	0.9021 (3)	0.8583 (11)	0.3213 (5)	0.0329 (9)	0.50
H25'	0.9004	0.8364	0.3576	0.039*	0.50
C26'	0.9282 (3)	1.0474 (11)	0.3304 (4)	0.0375 (16)	0.50
H26C	0.9188	1.1072	0.2950	0.045*	0.50
H26D	0.9126	1.1169	0.3530	0.045*	0.50
C27'	0.9963 (3)	1.0124 (12)	0.3613 (4)	0.0450 (17)	0.50
H27C	1.0112	1.0570	0.3995	0.054*	0.50
H27D	1.0188	1.0710	0.3425	0.054*	0.50
C28'	1.0058 (6)	0.8123 (13)	0.3618 (6)	0.0421 (13)	0.50
H28C	1.0389	0.7862	0.3505	0.051*	0.50
H28D	1.0173	0.7673	0.3996	0.051*	0.50
C29'	0.9489 (4)	0.7248 (13)	0.3231 (4)	0.0427 (12)	0.50
C30'	0.9486 (3)	0.6490 (10)	0.2694 (3)	0.0380 (14)	0.50
H30D	0.9702	0.5383	0.2773	0.057*	0.50
H30E	0.9686	0.7300	0.2540	0.057*	0.50
H30F	0.9069	0.6307	0.2428	0.057*	0.50
C31'	0.9282 (4)	0.5437 (11)	0.3375 (3)	0.0707 (16)	0.50
H31C	0.9222	0.5495	0.3721	0.085*	0.50
H31D	0.9576	0.4512	0.3407	0.085*	0.50
C32'	0.8661 (3)	0.5148 (12)	0.2856 (3)	0.0390 (17)	0.50
H32C	0.8708	0.5414	0.2512	0.047*	0.50
H32D	0.8528	0.3930	0.2840	0.047*	0.50

### Atomic displacement parameters (Å^2^)

**Table:**

	*U*^11^	*U*^22^	*U*^33^	*U*^12^	*U*^13^	*U*^23^
O1	0.0280 (11)	0.0275 (13)	0.0546 (15)	−0.0022 (10)	0.0159 (11)	−0.0072 (11)
O2	0.0234 (10)	0.0343 (14)	0.0390 (12)	−0.0058 (10)	0.0132 (9)	−0.0089 (11)
O3	0.0248 (11)	0.0269 (12)	0.0549 (15)	−0.0049 (10)	0.0176 (10)	−0.0080 (11)
O4	0.0363 (13)	0.0380 (15)	0.0503 (14)	−0.0099 (12)	0.0162 (11)	−0.0130 (13)
C1	0.0228 (15)	0.029 (4)	0.046 (3)	−0.001 (5)	0.020 (2)	−0.003 (4)
C2	0.026 (2)	0.022 (5)	0.032 (5)	0.005 (3)	0.015 (3)	0.007 (3)
C3	0.026 (3)	0.022 (4)	0.031 (4)	0.006 (3)	0.019 (3)	0.009 (3)
C4	0.029 (2)	0.026 (3)	0.035 (4)	0.006 (2)	0.013 (3)	0.006 (2)
C5	0.047 (3)	0.022 (3)	0.035 (3)	0.007 (3)	0.017 (3)	−0.0009 (19)
C6	0.035 (3)	0.020 (3)	0.027 (2)	0.006 (3)	0.013 (2)	−0.002 (2)
C7	0.044 (3)	0.033 (3)	0.023 (2)	0.004 (3)	0.011 (2)	−0.002 (2)
C8	0.058 (4)	0.038 (3)	0.039 (3)	−0.006 (3)	0.003 (3)	−0.004 (3)
C9	0.075 (5)	0.052 (4)	0.044 (3)	−0.011 (4)	−0.001 (3)	−0.007 (3)
C10	0.057 (4)	0.053 (4)	0.027 (2)	0.002 (3)	0.006 (2)	0.000 (3)
C11	0.034 (3)	0.039 (3)	0.0219 (19)	0.004 (2)	0.0085 (18)	0.002 (2)
C12	0.037 (3)	0.048 (5)	0.036 (2)	−0.002 (4)	0.013 (2)	0.004 (4)
C13	0.047 (3)	0.038 (3)	0.033 (2)	0.005 (2)	0.018 (2)	0.005 (2)
C14	0.041 (3)	0.025 (3)	0.040 (2)	0.005 (2)	0.022 (2)	0.010 (3)
C15	0.038 (3)	0.023 (3)	0.035 (2)	0.004 (3)	0.023 (2)	0.003 (3)
C16	0.034 (3)	0.014 (4)	0.036 (2)	0.002 (3)	0.023 (2)	0.008 (3)
C17	0.038 (3)	0.020 (4)	0.045 (2)	0.003 (3)	0.026 (2)	0.003 (4)
C18	0.033 (3)	0.025 (6)	0.051 (3)	−0.001 (4)	0.030 (2)	−0.009 (3)
C1'	0.0228 (15)	0.029 (4)	0.046 (3)	−0.001 (5)	0.020 (2)	−0.003 (4)
C2'	0.026 (2)	0.022 (5)	0.032 (5)	0.005 (3)	0.015 (3)	0.007 (3)
C3'	0.026 (3)	0.022 (4)	0.031 (4)	0.006 (3)	0.019 (3)	0.009 (3)
C4'	0.029 (2)	0.026 (3)	0.035 (4)	0.006 (2)	0.013 (3)	0.006 (2)
C5'	0.047 (3)	0.022 (3)	0.035 (3)	0.007 (3)	0.017 (3)	−0.0009 (19)
C6'	0.035 (3)	0.020 (3)	0.027 (2)	0.006 (3)	0.013 (2)	−0.002 (2)
C7'	0.044 (3)	0.033 (3)	0.023 (2)	0.004 (3)	0.011 (2)	−0.002 (2)
C8'	0.058 (4)	0.038 (3)	0.039 (3)	−0.006 (3)	0.003 (3)	−0.004 (3)
C9'	0.075 (5)	0.052 (4)	0.044 (3)	−0.011 (4)	−0.001 (3)	−0.007 (3)
C10'	0.057 (4)	0.053 (4)	0.027 (2)	0.002 (3)	0.006 (2)	0.000 (3)
C11'	0.034 (3)	0.039 (3)	0.0219 (19)	0.004 (2)	0.0085 (18)	0.002 (2)
C12'	0.037 (3)	0.048 (5)	0.036 (2)	−0.002 (4)	0.013 (2)	0.004 (4)
C13'	0.047 (3)	0.038 (3)	0.033 (2)	0.005 (2)	0.018 (2)	0.005 (2)
C14'	0.041 (3)	0.025 (3)	0.040 (2)	0.005 (2)	0.022 (2)	0.010 (3)
C15'	0.038 (3)	0.023 (3)	0.035 (2)	0.004 (3)	0.023 (2)	0.003 (3)
C16'	0.034 (3)	0.014 (4)	0.036 (2)	0.002 (3)	0.023 (2)	0.008 (3)
C17'	0.038 (3)	0.020 (4)	0.045 (2)	0.003 (3)	0.026 (2)	0.003 (4)
C18'	0.033 (3)	0.025 (6)	0.051 (3)	−0.001 (4)	0.030 (2)	−0.009 (3)
C19	0.0209 (14)	0.0385 (19)	0.0377 (17)	−0.0043 (14)	0.0155 (13)	−0.0092 (15)
C20	0.0221 (14)	0.0287 (17)	0.0320 (15)	0.0014 (13)	0.0151 (12)	−0.0029 (13)
C21	0.0237 (14)	0.0288 (17)	0.0328 (15)	−0.0023 (13)	0.0175 (13)	−0.0068 (14)
C22	0.0232 (15)	0.0322 (19)	0.0373 (17)	−0.0026 (13)	0.0158 (13)	−0.0062 (14)
C23	0.0348 (18)	0.0276 (19)	0.050 (2)	−0.0033 (15)	0.0043 (16)	−0.0014 (17)
C24	0.0187 (14)	0.042 (2)	0.0257 (15)	−0.0045 (13)	0.0086 (12)	0.0033 (14)
C25	0.0247 (15)	0.044 (2)	0.026 (2)	−0.0055 (15)	0.0066 (13)	0.0100 (16)
C26	0.028 (2)	0.037 (4)	0.039 (4)	−0.007 (2)	0.006 (3)	−0.001 (3)
C27	0.028 (2)	0.055 (4)	0.047 (4)	−0.008 (3)	0.011 (3)	0.000 (3)
C28	0.0234 (16)	0.054 (4)	0.038 (2)	−0.005 (2)	0.0016 (15)	0.020 (3)
C29	0.0170 (17)	0.078 (3)	0.0282 (18)	−0.0030 (19)	0.0040 (15)	−0.008 (2)
C30	0.0261 (19)	0.057 (4)	0.034 (2)	0.014 (3)	0.0156 (17)	0.006 (3)
C31	0.0219 (18)	0.047 (3)	0.119 (4)	0.0046 (18)	0.004 (2)	−0.013 (3)
C32	0.0193 (19)	0.041 (3)	0.056 (5)	0.003 (2)	0.015 (3)	−0.006 (4)
C33	0.0183 (14)	0.0329 (19)	0.055 (2)	0.0016 (14)	0.0092 (14)	−0.0071 (16)
C34	0.0238 (16)	0.0318 (19)	0.059 (2)	0.0001 (14)	0.0176 (16)	−0.0104 (18)
C35	0.0274 (18)	0.033 (2)	0.091 (3)	0.0026 (16)	0.015 (2)	−0.019 (2)
C36	0.0276 (17)	0.032 (2)	0.077 (3)	−0.0046 (15)	0.0198 (18)	−0.022 (2)
C37	0.0226 (14)	0.0235 (16)	0.0339 (16)	−0.0038 (12)	0.0108 (12)	0.0013 (13)
C38	0.0227 (14)	0.0199 (15)	0.0408 (17)	0.0028 (12)	0.0162 (13)	0.0007 (13)
C39	0.0252 (15)	0.0191 (16)	0.0318 (15)	0.0010 (12)	0.0125 (12)	0.0005 (13)
C40	0.0266 (15)	0.0190 (16)	0.0471 (18)	0.0017 (13)	0.0181 (14)	−0.0022 (14)
C41	0.0249 (14)	0.0163 (14)	0.0369 (16)	−0.0017 (12)	0.0140 (13)	−0.0023 (13)
C42	0.0221 (13)	0.0170 (14)	0.0273 (14)	0.0000 (11)	0.0122 (11)	0.0005 (12)
C43	0.0252 (14)	0.0209 (16)	0.0303 (15)	0.0004 (12)	0.0155 (12)	0.0012 (12)
C44	0.0335 (17)	0.0245 (17)	0.0431 (18)	−0.0040 (14)	0.0157 (15)	−0.0025 (15)
C45	0.0331 (17)	0.035 (2)	0.0456 (19)	−0.0100 (15)	0.0169 (15)	−0.0031 (16)
C46	0.0234 (14)	0.0349 (19)	0.0388 (17)	−0.0049 (14)	0.0129 (13)	0.0036 (15)
C47	0.0237 (14)	0.0248 (16)	0.0323 (16)	−0.0008 (12)	0.0134 (13)	0.0028 (13)
C48	0.0303 (16)	0.0347 (19)	0.0306 (16)	−0.0021 (14)	0.0090 (14)	0.0073 (14)
C49	0.0223 (14)	0.0271 (17)	0.0424 (17)	0.0044 (13)	0.0166 (13)	0.0032 (14)
C50	0.0239 (14)	0.0178 (15)	0.0406 (17)	0.0035 (12)	0.0155 (13)	0.0025 (13)
C51	0.0227 (13)	0.0151 (14)	0.0293 (14)	0.0010 (11)	0.0141 (11)	0.0000 (12)
C52	0.0196 (13)	0.0202 (15)	0.0252 (14)	0.0006 (11)	0.0099 (11)	0.0037 (12)
C53	0.0248 (14)	0.0227 (16)	0.0250 (14)	0.0029 (12)	0.0119 (11)	−0.0011 (12)
C54	0.0315 (15)	0.0188 (15)	0.0287 (15)	−0.0010 (12)	0.0131 (12)	−0.0015 (12)
C55	0.0352 (19)	0.068 (3)	0.056 (2)	−0.008 (2)	0.0183 (18)	−0.021 (2)
C25'	0.0247 (15)	0.044 (2)	0.026 (2)	−0.0055 (15)	0.0066 (13)	0.0100 (16)
C26'	0.028 (2)	0.037 (4)	0.039 (4)	−0.007 (2)	0.006 (3)	−0.001 (3)
C27'	0.028 (2)	0.055 (4)	0.047 (4)	−0.008 (3)	0.011 (3)	0.000 (3)
C28'	0.0234 (16)	0.054 (4)	0.038 (2)	−0.005 (2)	0.0016 (15)	0.020 (3)
C29'	0.0170 (17)	0.078 (3)	0.0282 (18)	−0.0030 (19)	0.0040 (15)	−0.008 (2)
C30'	0.0261 (19)	0.057 (4)	0.034 (2)	0.014 (3)	0.0156 (17)	0.006 (3)
C31'	0.0219 (18)	0.047 (3)	0.119 (4)	0.0046 (18)	0.004 (2)	−0.013 (3)
C32'	0.0193 (19)	0.041 (3)	0.056 (5)	0.003 (2)	0.015 (3)	−0.006 (4)

### Geometric parameters (Å, °)

**Table:**

O1—C1'	1.38 (2)	C23—H23A	0.9900
O1—C1	1.39 (2)	C23—H23B	0.9900
O1—H1	0.8400	C24—C33	1.507 (5)
O1—H1'	0.8400	C24—C25'	1.520 (8)
O2—C19	1.382 (4)	C24—C25	1.530 (8)
O2—H2	0.8400	C24—H24	1.0000
O3—C37	1.378 (4)	C24—H24'	1.0000
O3—H3	0.8400	C25—C26	1.549 (9)
O4—C55	1.402 (4)	C25—C29	1.583 (8)
O4—H4	0.8400	C25—H25	1.0000
C1—C2	1.386 (9)	C26—C27	1.571 (7)
C1—C18	1.388 (10)	C26—H26A	0.9900
C2—C3	1.391 (9)	C26—H26B	0.9900
C2—H2A	0.9500	C27—C28	1.595 (9)
C3—C16	1.394 (7)	C27—H27A	0.9900
C3—C4	1.522 (8)	C27—H27B	0.9900
C4—C5	1.532 (7)	C28—C29	1.598 (8)
C4—H4A	0.9900	C28—H28A	0.9900
C4—H4B	0.9900	C28—H28B	0.9900
C5—C6	1.523 (7)	C29—C31	1.352 (11)
C5—H5A	0.9900	C29—C30	1.603 (9)
C5—H5B	0.9900	C30—H30A	0.9800
C6—C7	1.529 (7)	C30—H30B	0.9800
C6—C15	1.536 (7)	C30—H30C	0.9800
C6—H6	1.0000	C31—C32	1.554 (7)
C7—C8	1.526 (8)	C31—H31A	0.9900
C7—C11	1.536 (8)	C31—H31B	0.9900
C7—H7	1.0000	C32—C33	1.503 (6)
C8—C9	1.542 (8)	C32—H32A	0.9900
C8—H8A	0.9900	C32—H32B	0.9900
C8—H8B	0.9900	C33—C34	1.523 (4)
C9—C10	1.557 (9)	C33—C32'	1.588 (7)
C9—H9A	0.9900	C33—H33	1.0000
C9—H9B	0.9900	C33—H33'	1.0000
C10—C11	1.539 (7)	C34—C35	1.399 (5)
C10—H10A	0.9900	C35—C36	1.382 (5)
C10—H10B	0.9900	C35—H35	0.9500
C11—C12	1.535 (8)	C36—H36	0.9500
C11—C13	1.542 (8)	C37—C38	1.380 (5)
C12—H12A	0.9800	C37—C54	1.389 (4)
C12—H12B	0.9800	C38—C39	1.395 (4)
C12—H12C	0.9800	C38—H38	0.9500
C13—C14	1.550 (7)	C39—C52	1.401 (4)
C13—H13A	0.9900	C39—C40	1.515 (4)
C13—H13B	0.9900	C40—C41	1.527 (4)
C14—C15	1.532 (7)	C40—H40A	0.9900
C14—H14A	0.9900	C40—H40B	0.9900
C14—H14B	0.9900	C41—C42	1.517 (4)
C15—C16	1.536 (8)	C41—H41A	0.9900
C15—H15	1.0000	C41—H41B	0.9900
C16—C17	1.393 (8)	C42—C43	1.523 (4)
C17—C18	1.392 (9)	C42—C51	1.535 (4)
C17—H17	0.9500	C42—H42	1.0000
C18—H18A	0.9500	C43—C44	1.532 (4)
C1'—C18'	1.390 (10)	C43—C47	1.541 (4)
C1'—C2'	1.390 (9)	C43—H43	1.0000
C2'—C3'	1.392 (9)	C44—C45	1.557 (5)
C2'—H2'	0.9500	C44—H44A	0.9900
C3'—C16'	1.393 (7)	C44—H44B	0.9900
C3'—C4'	1.522 (8)	C45—C46	1.543 (5)
C4'—C5'	1.537 (7)	C45—H45A	0.9900
C4'—H4'A	0.9900	C45—H45B	0.9900
C4'—H4'B	0.9900	C46—C47	1.534 (4)
C5'—C6'	1.526 (7)	C46—H46A	0.9900
C5'—H5'A	0.9900	C46—H46B	0.9900
C5'—H5'B	0.9900	C47—C49	1.523 (5)
C6'—C15'	1.530 (7)	C47—C48	1.534 (4)
C6'—C7'	1.536 (7)	C48—H48A	0.9800
C6'—H6'	1.0000	C48—H48B	0.9800
C7'—C8'	1.532 (8)	C48—H48C	0.9800
C7'—C11'	1.535 (8)	C49—C50	1.534 (4)
C7'—H7'	1.0000	C49—H49A	0.9900
C8'—C9'	1.541 (8)	C49—H49B	0.9900
C8'—H8'A	0.9900	C50—C51	1.538 (4)
C8'—H8'B	0.9900	C50—H50A	0.9900
C9'—C10'	1.539 (9)	C50—H50B	0.9900
C9'—H9'1	0.9900	C51—C52	1.527 (4)
C9'—H9'2	0.9900	C51—H51	1.0000
C10'—C11'	1.542 (8)	C52—C53	1.400 (4)
C10'—H10C	0.9900	C53—C54	1.385 (4)
C10'—H10D	0.9900	C53—H53	0.9500
C11'—C13'	1.525 (8)	C54—H54	0.9500
C11'—C12'	1.536 (8)	C55—H55A	0.9800
C12'—H12D	0.9800	C55—H55B	0.9800
C12'—H12E	0.9800	C55—H55C	0.9800
C12'—H12F	0.9800	C25'—C29'	1.521 (7)
C13'—C14'	1.546 (8)	C25'—C26'	1.571 (8)
C13'—H13C	0.9900	C25'—H25'	1.0000
C13'—H13D	0.9900	C26'—C27'	1.540 (8)
C14'—C15'	1.535 (7)	C26'—H26C	0.9900
C14'—H14C	0.9900	C26'—H26D	0.9900
C14'—H14D	0.9900	C27'—C28'	1.562 (9)
C15'—C16'	1.548 (8)	C27'—H27C	0.9900
C15'—H15'	1.0000	C27'—H27D	0.9900
C16'—C17'	1.387 (8)	C28'—C29'	1.503 (9)
C17'—C18'	1.393 (9)	C28'—H28C	0.9900
C17'—H17'	0.9500	C28'—H28D	0.9900
C18'—H18'	0.9500	C29'—C30'	1.546 (8)
C19—C20	1.382 (5)	C29'—C31'	1.586 (14)
C19—C36	1.383 (5)	C30'—H30D	0.9800
C20—C21	1.389 (4)	C30'—H30E	0.9800
C20—H20	0.9500	C30'—H30F	0.9800
C21—C34	1.411 (5)	C31'—C32'	1.589 (8)
C21—C22	1.506 (5)	C31'—H31C	0.9900
C22—C23	1.510 (4)	C31'—H31D	0.9900
C22—H22A	0.9900	C32'—H32C	0.9900
C22—H22B	0.9900	C32'—H32D	0.9900
C23—C24	1.531 (5)		
			
C1—O1—H1	120.0	C25'—C24—H24	104.8
C1'—O1—H1'	120.2	C25—C24—H24	109.7
C1—O1—H1'	109.5	C23—C24—H24	109.7
C19—O2—H2	109.5	C33—C24—H24'	108.0
C37—O3—H3	109.5	C25'—C24—H24'	108.0
C55—O4—H4	109.5	C25—C24—H24'	112.9
C2—C1—C18	118.6 (17)	C23—C24—H24'	108.0
C2—C1—O1	116.5 (11)	C24—C25—C26	113.5 (7)
C18—C1—O1	124.5 (12)	C24—C25—C29	107.7 (6)
C1—C2—C3	122.7 (12)	C24—C25—H25	113.1
C1—C2—H2A	118.7	C26—C25—H25	113.1
C3—C2—H2A	118.7	C29—C25—H25	113.1
C2—C3—C16	118.5 (7)	C25—C26—C27	107.5 (5)
C2—C3—C4	116.8 (7)	C25—C26—H26A	110.2
C16—C3—C4	124.6 (7)	C27—C26—H26A	110.2
C3—C4—C5	112.4 (6)	C25—C26—H26B	110.2
C3—C4—H4A	109.1	C27—C26—H26B	110.2
C5—C4—H4A	109.1	H26A—C26—H26B	108.5
C3—C4—H4B	109.1	C26—C27—C28	103.3 (6)
C5—C4—H4B	109.1	C26—C27—H27A	111.1
H4A—C4—H4B	107.8	C28—C27—H27A	111.1
C6—C5—C4	111.9 (5)	C26—C27—H27B	111.1
C6—C5—H5A	109.2	C28—C27—H27B	111.1
C4—C5—H5A	109.2	H27A—C27—H27B	109.1
C6—C5—H5B	109.2	C27—C28—C29	97.9 (5)
C4—C5—H5B	109.2	C27—C28—H28A	112.2
H5A—C5—H5B	107.9	C29—C28—H28A	112.2
C5—C6—C7	111.9 (5)	C27—C28—H28B	112.2
C5—C6—C15	108.2 (5)	C29—C28—H28B	112.2
C7—C6—C15	109.9 (5)	H28A—C28—H28B	109.8
C5—C6—H6	108.9	C31—C29—C25	111.9 (6)
C7—C6—H6	108.9	C31—C29—C28	114.0 (7)
C15—C6—H6	108.9	C25—C29—C28	98.7 (8)
C8—C7—C6	119.6 (6)	C31—C29—C30	125.5 (7)
C8—C7—C11	105.4 (6)	C25—C29—C30	102.2 (6)
C6—C7—C11	112.6 (5)	C28—C29—C30	100.6 (7)
C8—C7—H7	106.1	C29—C31—C32	119.7 (7)
C6—C7—H7	106.1	C29—C31—H31A	107.4
C11—C7—H7	106.1	C32—C31—H31A	107.4
C7—C8—C9	103.4 (6)	C29—C31—H31B	107.4
C7—C8—H8A	111.1	C32—C31—H31B	107.4
C9—C8—H8A	111.1	H31A—C31—H31B	106.9
C7—C8—H8B	111.1	C33—C32—C31	113.2 (5)
C9—C8—H8B	111.1	C33—C32—H32A	108.9
H8A—C8—H8B	109.0	C31—C32—H32A	108.9
C8—C9—C10	106.2 (7)	C33—C32—H32B	108.9
C8—C9—H9A	110.5	C31—C32—H32B	108.9
C10—C9—H9A	110.5	H32A—C32—H32B	107.7
C8—C9—H9B	110.5	C32—C33—C24	121.4 (3)
C10—C9—H9B	110.5	C32—C33—C34	116.0 (4)
H9A—C9—H9B	108.7	C24—C33—C34	110.3 (3)
C11—C10—C9	104.8 (6)	C24—C33—C32'	104.5 (4)
C11—C10—H10A	110.8	C34—C33—C32'	111.1 (4)
C9—C10—H10A	110.8	C32—C33—H33	101.8
C11—C10—H10B	110.8	C24—C33—H33	101.8
C9—C10—H10B	110.8	C34—C33—H33	101.8
H10A—C10—H10B	108.9	C24—C33—H33'	110.3
C12—C11—C7	113.6 (6)	C34—C33—H33'	110.3
C12—C11—C10	108.9 (6)	C32'—C33—H33'	110.3
C7—C11—C10	99.0 (6)	C35—C34—C21	117.4 (3)
C12—C11—C13	110.5 (6)	C35—C34—C33	122.8 (3)
C7—C11—C13	108.3 (5)	C21—C34—C33	119.8 (3)
C10—C11—C13	116.2 (5)	C36—C35—C34	122.8 (4)
C11—C13—C14	111.3 (5)	C36—C35—H35	118.6
C11—C13—H13A	109.4	C34—C35—H35	118.6
C14—C13—H13A	109.4	C35—C36—C19	118.7 (3)
C11—C13—H13B	109.4	C35—C36—H36	120.7
C14—C13—H13B	109.4	C19—C36—H36	120.7
H13A—C13—H13B	108.0	O3—C37—C38	121.6 (3)
C15—C14—C13	113.5 (6)	O3—C37—C54	118.5 (3)
C15—C14—H14A	108.9	C38—C37—C54	119.9 (3)
C13—C14—H14A	108.9	C37—C38—C39	121.1 (3)
C15—C14—H14B	108.9	C37—C38—H38	119.4
C13—C14—H14B	108.9	C39—C38—H38	119.4
H14A—C14—H14B	107.7	C38—C39—C52	119.9 (3)
C14—C15—C6	111.8 (5)	C38—C39—C40	118.0 (3)
C14—C15—C16	113.2 (5)	C52—C39—C40	122.1 (3)
C6—C15—C16	111.1 (5)	C39—C40—C41	112.1 (3)
C14—C15—H15	106.8	C39—C40—H40A	109.2
C6—C15—H15	106.8	C41—C40—H40A	109.2
C16—C15—H15	106.8	C39—C40—H40B	109.2
C17—C16—C3	118.9 (7)	C41—C40—H40B	109.2
C17—C16—C15	122.3 (6)	H40A—C40—H40B	107.9
C3—C16—C15	118.8 (6)	C42—C41—C40	108.5 (3)
C18—C17—C16	121.9 (9)	C42—C41—H41A	110.0
C18—C17—H17	119.1	C40—C41—H41A	110.0
C16—C17—H17	119.1	C42—C41—H41B	110.0
C1—C18—C17	119.2 (13)	C40—C41—H41B	110.0
C1—C18—H18A	120.4	H41A—C41—H41B	108.4
C17—C18—H18A	120.4	C41—C42—C43	114.9 (3)
O1—C1'—C18'	119.9 (12)	C41—C42—C51	109.1 (2)
O1—C1'—C2'	118.1 (12)	C43—C42—C51	107.8 (2)
C18'—C1'—C2'	121.6 (16)	C41—C42—H42	108.3
C1'—C2'—C3'	119.2 (11)	C43—C42—H42	108.3
C1'—C2'—H2'	120.4	C51—C42—H42	108.3
C3'—C2'—H2'	120.4	C42—C43—C44	119.5 (3)
C2'—C3'—C16'	120.6 (7)	C42—C43—C47	112.6 (2)
C2'—C3'—C4'	116.4 (7)	C44—C43—C47	103.8 (2)
C16'—C3'—C4'	122.9 (7)	C42—C43—H43	106.7
C3'—C4'—C5'	113.9 (6)	C44—C43—H43	106.7
C3'—C4'—H4'A	108.8	C47—C43—H43	106.7
C5'—C4'—H4'A	108.8	C43—C44—C45	103.4 (3)
C3'—C4'—H4'B	108.8	C43—C44—H44A	111.1
C5'—C4'—H4'B	108.8	C45—C44—H44A	111.1
H4'A—C4'—H4'B	107.7	C43—C44—H44B	111.1
C6'—C5'—C4'	110.2 (5)	C45—C44—H44B	111.1
C6'—C5'—H5'A	109.6	H44A—C44—H44B	109.0
C4'—C5'—H5'A	109.6	C46—C45—C44	106.3 (3)
C6'—C5'—H5'B	109.6	C46—C45—H45A	110.5
C4'—C5'—H5'B	109.6	C44—C45—H45A	110.5
H5'A—C5'—H5'B	108.1	C46—C45—H45B	110.5
C5'—C6'—C15'	109.7 (5)	C44—C45—H45B	110.5
C5'—C6'—C7'	112.1 (5)	H45A—C45—H45B	108.7
C15'—C6'—C7'	109.1 (5)	C47—C46—C45	104.9 (3)
C5'—C6'—H6'	108.6	C47—C46—H46A	110.8
C15'—C6'—H6'	108.6	C45—C46—H46A	110.8
C7'—C6'—H6'	108.6	C47—C46—H46B	110.8
C8'—C7'—C11'	104.2 (6)	C45—C46—H46B	110.8
C8'—C7'—C6'	119.0 (5)	H46A—C46—H46B	108.8
C11'—C7'—C6'	113.1 (5)	C49—C47—C48	110.8 (3)
C8'—C7'—H7'	106.6	C49—C47—C46	116.6 (3)
C11'—C7'—H7'	106.6	C48—C47—C46	108.3 (3)
C6'—C7'—H7'	106.6	C49—C47—C43	108.4 (2)
C7'—C8'—C9'	103.1 (6)	C48—C47—C43	111.9 (3)
C7'—C8'—H8'A	111.1	C46—C47—C43	100.3 (3)
C9'—C8'—H8'A	111.1	C47—C48—H48A	109.5
C7'—C8'—H8'B	111.1	C47—C48—H48B	109.5
C9'—C8'—H8'B	111.1	H48A—C48—H48B	109.5
H8'A—C8'—H8'B	109.1	C47—C48—H48C	109.5
C10'—C9'—C8'	106.5 (7)	H48A—C48—H48C	109.5
C10'—C9'—H9'1	110.4	H48B—C48—H48C	109.5
C8'—C9'—H9'1	110.4	C47—C49—C50	112.5 (3)
C10'—C9'—H9'2	110.4	C47—C49—H49A	109.1
C8'—C9'—H9'2	110.4	C50—C49—H49A	109.1
H9'1—C9'—H9'2	108.6	C47—C49—H49B	109.1
C9'—C10'—C11'	105.0 (6)	C50—C49—H49B	109.1
C9'—C10'—H10C	110.7	H49A—C49—H49B	107.8
C11'—C10'—H10C	110.7	C49—C50—C51	112.7 (3)
C9'—C10'—H10D	110.7	C49—C50—H50A	109.0
C11'—C10'—H10D	110.7	C51—C50—H50A	109.0
H10C—C10'—H10D	108.8	C49—C50—H50B	109.0
C13'—C11'—C7'	107.6 (6)	C51—C50—H50B	109.0
C13'—C11'—C12'	109.8 (6)	H50A—C50—H50B	107.8
C7'—C11'—C12'	113.1 (6)	C52—C51—C42	111.8 (2)
C13'—C11'—C10'	117.0 (6)	C52—C51—C50	113.2 (2)
C7'—C11'—C10'	99.3 (6)	C42—C51—C50	111.3 (2)
C12'—C11'—C10'	109.6 (7)	C52—C51—H51	106.7
C11'—C12'—H12D	109.5	C42—C51—H51	106.7
C11'—C12'—H12E	109.5	C50—C51—H51	106.7
H12D—C12'—H12E	109.5	C53—C52—C39	117.7 (3)
C11'—C12'—H12F	109.5	C53—C52—C51	121.4 (3)
H12D—C12'—H12F	109.5	C39—C52—C51	120.9 (3)
H12E—C12'—H12F	109.5	C54—C53—C52	122.3 (3)
C11'—C13'—C14'	112.3 (5)	C54—C53—H53	118.9
C11'—C13'—H13C	109.1	C52—C53—H53	118.9
C14'—C13'—H13C	109.1	C53—C54—C37	119.0 (3)
C11'—C13'—H13D	109.1	C53—C54—H54	120.5
C14'—C13'—H13D	109.1	C37—C54—H54	120.5
H13C—C13'—H13D	107.9	O4—C55—H55A	109.5
C15'—C14'—C13'	112.1 (6)	O4—C55—H55B	109.5
C15'—C14'—H14C	109.2	H55A—C55—H55B	109.5
C13'—C14'—H14C	109.2	O4—C55—H55C	109.5
C15'—C14'—H14D	109.2	H55A—C55—H55C	109.5
C13'—C14'—H14D	109.2	H55B—C55—H55C	109.5
H14C—C14'—H14D	107.9	C24—C25'—C29'	116.2 (7)
C6'—C15'—C14'	112.5 (5)	C24—C25'—C26'	122.0 (6)
C6'—C15'—C16'	109.6 (5)	C29'—C25'—C26'	112.1 (7)
C14'—C15'—C16'	113.0 (5)	C24—C25'—H25'	100.5
C6'—C15'—H15'	107.1	C29'—C25'—H25'	100.5
C14'—C15'—H15'	107.1	C26'—C25'—H25'	100.5
C16'—C15'—H15'	107.1	C27'—C26'—C25'	101.4 (6)
C17'—C16'—C3'	118.2 (7)	C27'—C26'—H26C	111.5
C17'—C16'—C15'	121.3 (6)	C25'—C26'—H26C	111.5
C3'—C16'—C15'	120.4 (6)	C27'—C26'—H26D	111.5
C16'—C17'—C18'	122.7 (9)	C25'—C26'—H26D	111.5
C16'—C17'—H17'	118.7	H26C—C26'—H26D	109.3
C18'—C17'—H17'	118.7	C26'—C27'—C28'	107.5 (7)
C1'—C18'—C17'	117.3 (14)	C26'—C27'—H27C	110.2
C1'—C18'—H18'	121.4	C28'—C27'—H27C	110.2
C17'—C18'—H18'	121.4	C26'—C27'—H27D	110.2
O2—C19—C20	118.1 (3)	C28'—C27'—H27D	110.2
O2—C19—C36	121.7 (3)	H27C—C27'—H27D	108.5
C20—C19—C36	120.2 (3)	C29'—C28'—C27'	110.4 (8)
C19—C20—C21	121.3 (3)	C29'—C28'—H28C	109.6
C19—C20—H20	119.4	C27'—C28'—H28C	109.6
C21—C20—H20	119.4	C29'—C28'—H28D	109.6
C20—C21—C34	119.5 (3)	C27'—C28'—H28D	109.6
C20—C21—C22	118.7 (3)	H28C—C28'—H28D	108.1
C34—C21—C22	121.7 (3)	C28'—C29'—C25'	100.5 (9)
C21—C22—C23	114.5 (3)	C28'—C29'—C30'	117.2 (9)
C21—C22—H22A	108.6	C25'—C29'—C30'	120.6 (8)
C23—C22—H22A	108.6	C28'—C29'—C31'	121.7 (8)
C21—C22—H22B	108.6	C25'—C29'—C31'	107.3 (7)
C23—C22—H22B	108.6	C30'—C29'—C31'	90.5 (6)
H22A—C22—H22B	107.6	C29'—C31'—C32'	101.3 (7)
C22—C23—C24	113.2 (3)	C29'—C31'—H31C	111.5
C22—C23—H23A	108.9	C32'—C31'—H31C	111.5
C24—C23—H23A	108.9	C29'—C31'—H31D	111.5
C22—C23—H23B	108.9	C32'—C31'—H31D	111.5
C24—C23—H23B	108.9	H31C—C31'—H31D	109.3
H23A—C23—H23B	107.8	C33—C32'—C31'	107.0 (6)
C33—C24—C25'	111.6 (5)	C33—C32'—H32C	110.3
C33—C24—C25	108.8 (4)	C31'—C32'—H32C	110.3
C33—C24—C23	107.9 (3)	C33—C32'—H32D	110.3
C25'—C24—C23	113.1 (4)	C31'—C32'—H32D	110.3
C25—C24—C23	111.1 (3)	H32C—C32'—H32D	108.6
C33—C24—H24	109.7		
			
C1'—O1—C1—C2	10 (21)	C29—C25—C26—C27	−40.4 (8)
C1'—O1—C1—C18	−163 (30)	C25—C26—C27—C28	7.4 (11)
C18—C1—C2—C3	−2(6)	C26—C27—C28—C29	28.7 (12)
O1—C1—C2—C3	−176 (3)	C24—C25—C29—C31	−64.6 (10)
C1—C2—C3—C16	2(4)	C26—C25—C29—C31	178.8 (7)
C1—C2—C3—C4	−179 (3)	C24—C25—C29—C28	175.0 (8)
C2—C3—C4—C5	176.8 (12)	C26—C25—C29—C28	58.4 (8)
C16—C3—C4—C5	−4.4 (14)	C24—C25—C29—C30	72.1 (7)
C3—C4—C5—C6	37.6 (10)	C26—C25—C29—C30	−44.5 (7)
C4—C5—C6—C7	174.0 (6)	C27—C28—C29—C31	−173.7 (9)
C4—C5—C6—C15	−64.8 (7)	C27—C28—C29—C25	−54.9 (11)
C5—C6—C7—C8	−56.1 (9)	C27—C28—C29—C30	49.4 (11)
C15—C6—C7—C8	−176.3 (7)	C25—C29—C31—C32	46.5 (12)
C5—C6—C7—C11	179.4 (6)	C28—C29—C31—C32	157.5 (11)
C15—C6—C7—C11	59.2 (7)	C30—C29—C31—C32	−78.1 (11)
C6—C7—C8—C9	−162.0 (7)	C29—C31—C32—C33	−23.7 (13)
C11—C7—C8—C9	−34.1 (9)	C31—C32—C33—C24	19.7 (9)
C7—C8—C9—C10	8.1 (11)	C31—C32—C33—C34	158.3 (7)
C8—C9—C10—C11	20.3 (11)	C31—C32—C33—C32'	73.4 (12)
C8—C7—C11—C12	−69.1 (7)	C25'—C24—C33—C32	−35.2 (7)
C6—C7—C11—C12	62.9 (8)	C25—C24—C33—C32	−39.4 (7)
C8—C7—C11—C10	46.1 (7)	C23—C24—C33—C32	−160.0 (5)
C6—C7—C11—C10	178.1 (6)	C25'—C24—C33—C34	−175.9 (5)
C8—C7—C11—C13	167.7 (6)	C25—C24—C33—C34	179.9 (4)
C6—C7—C11—C13	−60.3 (7)	C23—C24—C33—C34	59.3 (4)
C9—C10—C11—C12	79.0 (9)	C25'—C24—C33—C32'	−56.4 (6)
C9—C10—C11—C7	−39.8 (9)	C25—C24—C33—C32'	−60.6 (5)
C9—C10—C11—C13	−155.5 (8)	C23—C24—C33—C32'	178.8 (4)
C12—C11—C13—C14	−69.6 (8)	C20—C21—C34—C35	3.8 (5)
C7—C11—C13—C14	55.4 (7)	C22—C21—C34—C35	−173.6 (4)
C10—C11—C13—C14	165.7 (6)	C20—C21—C34—C33	−175.1 (3)
C11—C13—C14—C15	−52.5 (8)	C22—C21—C34—C33	7.4 (5)
C13—C14—C15—C6	50.7 (7)	C32—C33—C34—C35	4.0 (7)
C13—C14—C15—C16	177.1 (6)	C24—C33—C34—C35	147.0 (4)
C5—C6—C15—C14	−175.2 (5)	C32'—C33—C34—C35	31.6 (7)
C7—C6—C15—C14	−52.8 (7)	C32—C33—C34—C21	−177.1 (5)
C5—C6—C15—C16	57.3 (7)	C24—C33—C34—C21	−34.1 (5)
C7—C6—C15—C16	179.7 (6)	C32'—C33—C34—C21	−149.5 (5)
C2—C3—C16—C17	−2.6 (16)	C21—C34—C35—C36	−3.6 (7)
C4—C3—C16—C17	178.6 (9)	C33—C34—C35—C36	175.4 (4)
C2—C3—C16—C15	177.3 (12)	C34—C35—C36—C19	0.5 (7)
C4—C3—C16—C15	−1.5 (14)	O2—C19—C36—C35	−176.5 (4)
C14—C15—C16—C17	27.7 (10)	C20—C19—C36—C35	2.3 (6)
C6—C15—C16—C17	154.5 (7)	O3—C37—C38—C39	178.9 (3)
C14—C15—C16—C3	−152.2 (8)	C54—C37—C38—C39	0.2 (5)
C6—C15—C16—C3	−25.4 (10)	C37—C38—C39—C52	2.0 (5)
C3—C16—C17—C18	3.3 (15)	C37—C38—C39—C40	−176.5 (3)
C15—C16—C17—C18	−176.6 (10)	C38—C39—C40—C41	163.0 (3)
C2—C1—C18—C17	3(5)	C52—C39—C40—C41	−15.5 (4)
O1—C1—C18—C17	175 (3)	C39—C40—C41—C42	50.1 (3)
C16—C17—C18—C1	−3(3)	C40—C41—C42—C43	170.2 (3)
C1—O1—C1'—C18'	0(21)	C40—C41—C42—C51	−68.7 (3)
C1—O1—C1'—C2'	−173 (29)	C41—C42—C43—C44	−54.2 (4)
O1—C1'—C2'—C3'	179 (3)	C51—C42—C43—C44	−176.0 (3)
C18'—C1'—C2'—C3'	6(6)	C41—C42—C43—C47	−176.4 (3)
C1'—C2'—C3'—C16'	−1(4)	C51—C42—C43—C47	61.8 (3)
C1'—C2'—C3'—C4'	178 (3)	C42—C43—C44—C45	−161.9 (3)
C2'—C3'—C4'—C5'	174.3 (12)	C47—C43—C44—C45	−35.5 (3)
C16'—C3'—C4'—C5'	−6.3 (13)	C43—C44—C45—C46	10.9 (4)
C3'—C4'—C5'—C6'	38.3 (9)	C44—C45—C46—C47	17.7 (4)
C4'—C5'—C6'—C15'	−65.4 (7)	C45—C46—C47—C49	−155.7 (3)
C4'—C5'—C6'—C7'	173.2 (6)	C45—C46—C47—C48	78.5 (3)
C5'—C6'—C7'—C8'	−56.8 (9)	C45—C46—C47—C43	−38.9 (3)
C15'—C6'—C7'—C8'	−178.5 (6)	C42—C43—C47—C49	−60.2 (3)
C5'—C6'—C7'—C11'	−179.5 (6)	C44—C43—C47—C49	169.1 (3)
C15'—C6'—C7'—C11'	58.8 (7)	C42—C43—C47—C48	62.3 (3)
C11'—C7'—C8'—C9'	−36.5 (8)	C44—C43—C47—C48	−68.3 (3)
C6'—C7'—C8'—C9'	−163.6 (7)	C42—C43—C47—C46	177.0 (3)
C7'—C8'—C9'—C10'	11.3 (10)	C44—C43—C47—C46	46.4 (3)
C8'—C9'—C10'—C11'	17.7 (11)	C48—C47—C49—C50	−69.8 (3)
C8'—C7'—C11'—C13'	169.2 (6)	C46—C47—C49—C50	165.7 (3)
C6'—C7'—C11'—C13'	−60.2 (7)	C43—C47—C49—C50	53.4 (3)
C8'—C7'—C11'—C12'	−69.3 (8)	C47—C49—C50—C51	−51.7 (4)
C6'—C7'—C11'—C12'	61.4 (8)	C41—C42—C51—C52	50.2 (3)
C8'—C7'—C11'—C10'	46.8 (7)	C43—C42—C51—C52	175.6 (2)
C6'—C7'—C11'—C10'	177.5 (6)	C41—C42—C51—C50	177.9 (2)
C9'—C10'—C11'—C13'	−154.4 (8)	C43—C42—C51—C50	−56.7 (3)
C9'—C10'—C11'—C7'	−39.0 (9)	C49—C50—C51—C52	−180.0 (3)
C9'—C10'—C11'—C12'	79.7 (9)	C49—C50—C51—C42	53.1 (3)
C7'—C11'—C13'—C14'	56.3 (7)	C38—C39—C52—C53	−2.9 (4)
C12'—C11'—C13'—C14'	−67.3 (8)	C40—C39—C52—C53	175.6 (3)
C10'—C11'—C13'—C14'	166.9 (7)	C38—C39—C52—C51	179.3 (3)
C11'—C13'—C14'—C15'	−53.6 (8)	C40—C39—C52—C51	−2.2 (4)
C5'—C6'—C15'—C14'	−176.1 (5)	C42—C51—C52—C53	167.0 (2)
C7'—C6'—C15'—C14'	−53.0 (7)	C50—C51—C52—C53	40.2 (4)
C5'—C6'—C15'—C16'	57.3 (7)	C42—C51—C52—C39	−15.3 (4)
C7'—C6'—C15'—C16'	−179.5 (6)	C50—C51—C52—C39	−142.0 (3)
C13'—C14'—C15'—C6'	51.4 (7)	C39—C52—C53—C54	1.7 (4)
C13'—C14'—C15'—C16'	176.2 (6)	C51—C52—C53—C54	179.5 (3)
C2'—C3'—C16'—C17'	−3.2 (18)	C52—C53—C54—C37	0.5 (5)
C4'—C3'—C16'—C17'	177.4 (8)	O3—C37—C54—C53	179.8 (3)
C2'—C3'—C16'—C15'	179.0 (12)	C38—C37—C54—C53	−1.5 (5)
C4'—C3'—C16'—C15'	−0.4 (15)	C33—C24—C25'—C29'	52.5 (11)
C6'—C15'—C16'—C17'	157.2 (7)	C25—C24—C25'—C29'	107 (6)
C14'—C15'—C16'—C17'	30.9 (10)	C23—C24—C25'—C29'	174.4 (8)
C6'—C15'—C16'—C3'	−25.0 (11)	C33—C24—C25'—C26'	−164.2 (8)
C14'—C15'—C16'—C3'	−151.3 (8)	C25—C24—C25'—C26'	−109 (6)
C3'—C16'—C17'—C18'	2.7 (15)	C23—C24—C25'—C26'	−42.3 (11)
C15'—C16'—C17'—C18'	−179.5 (9)	C24—C25'—C26'—C27'	−167.7 (9)
O1—C1'—C18'—C17'	−179 (3)	C29'—C25'—C26'—C27'	−23.1 (11)
C2'—C1'—C18'—C17'	−6(6)	C25'—C26'—C27'—C28'	6.6 (11)
C16'—C17'—C18'—C1'	2(3)	C26'—C27'—C28'—C29'	11.4 (16)
O2—C19—C20—C21	176.8 (3)	C27'—C28'—C29'—C25'	−24.2 (15)
C36—C19—C20—C21	−2.0 (5)	C27'—C28'—C29'—C30'	108.4 (13)
C19—C20—C21—C34	−1.2 (5)	C27'—C28'—C29'—C31'	−142.3 (9)
C19—C20—C21—C22	176.3 (3)	C24—C25'—C29'—C28'	176.4 (10)
C20—C21—C22—C23	175.0 (3)	C26'—C25'—C29'—C28'	29.6 (13)
C34—C21—C22—C23	−7.5 (5)	C24—C25'—C29'—C30'	45.9 (13)
C21—C22—C23—C24	34.8 (4)	C26'—C25'—C29'—C30'	−100.9 (10)
C22—C23—C24—C33	−61.7 (4)	C24—C25'—C29'—C31'	−55.4 (11)
C22—C23—C24—C25'	174.4 (6)	C26'—C25'—C29'—C31'	157.8 (8)
C22—C23—C24—C25	179.2 (5)	C28'—C29'—C31'—C32'	177.9 (10)
C33—C24—C25—C26	161.7 (5)	C25'—C29'—C31'—C32'	63.3 (7)
C25'—C24—C25—C26	35 (6)	C30'—C29'—C31'—C32'	−59.2 (6)
C23—C24—C25—C26	−79.7 (7)	C32—C33—C32'—C31'	−63.4 (10)
C33—C24—C25—C29	57.8 (7)	C24—C33—C32'—C31'	71.2 (6)
C25'—C24—C25—C29	−69 (6)	C34—C33—C32'—C31'	−169.8 (5)
C23—C24—C25—C29	176.5 (6)	C29'—C31'—C32'—C33	−74.4 (6)
C24—C25—C26—C27	−152.2 (6)		

### Hydrogen-bond geometry (Å, °)

**Table:**

*D*—H···*A*	*D*—H	H···*A*	*D*···*A*	*D*—H···*A*
O1—H1···O3	0.84	1.83	2.662 (3)	170
O2—H2···O4	0.84	1.82	2.655 (4)	170
O3—H3···O2	0.84	1.88	2.711 (3)	173
O4—H4···O1^i^	0.84	1.90	2.736 (4)	178

Symmetry codes: (i) *x*, *y*−1, *z*.
